# Supplementary material for: Antibacterial effects of thyme oil loaded solid lipid and chitosan nano-carriers against Salmonella Typhimurium and Escherichia coli as food preservatives
Source: PLoS One. 2024 Dec 31;19(12):e0315543. doi: 10.1371/journal.pone.0315543 (PMC12140078; doi:10.1371/journal.pone.0315543)
Supplement: S1 Table — (DOCX) [file pone.0315543.s001.docx]

**Table S1.** Absorption, concentration and cumulative oil release from TO-SLN in 72 h.

| Time (h) | Absorption 1 | Absorption 2 | Absorption 3 | Average | Concentration (mg/mL) | Cumulative drug release (%) |
| --- | --- | --- | --- | --- | --- | --- |
| 1 | 0.3247 | 0.3241 | 0.3228 | 0.3239 | 0.07028 | 1.4055±0.23 |
| 3 | 0.8972 | 0.9357 | 0.9876 | 0.94017 | 0.96031 | 19.2066±2.38 |
| 6 | 1.9121 | 1.8614 | 1.8179 | 1.8638 | 2.30144 | 46.0285±3.79 |
| 24 | 2.4396 | 2.4467 | 2.4413 | 2.4425 | 3.1418 | 62.8351±5.28 |
| 48 | 3.0097 | 2.9171 | 3.0285 | 2.9851 | 3.9296 | 78.5914±6.35 |
| 72 | 3.3287 | 3.3373 | 3.3276 | 3.3312 | 4.43.21 | 88.642±5.64 |
