# Supplementary material for: Antibacterial effects of thyme oil loaded solid lipid and chitosan nano-carriers against Salmonella Typhimurium and Escherichia coli as food preservatives
Source: PLoS One. 2024 Dec 31;19(12):e0315543. doi: 10.1371/journal.pone.0315543 (PMC12140078; doi:10.1371/journal.pone.0315543)
Supplement: S2 Table — (DOCX) [file pone.0315543.s002.docx]

**TableS2.** Absorption, concentration and cumulative oil release from TO-CH in 72 h.

| Time (h) | Absorption 1 | Absorption 2 | Absorption 3 | Average | Concentration (mg/mL) | Cumulative drug release (%) |
| --- | --- | --- | --- | --- | --- | --- |
| 1 | 0.5927 | 0.5942 | 0.6028 | 0.596567 | 0.461401 | 9.2280±1.35 |
| 3 | 1.4476 | 1.4374 | 1.4346 | 1.439867 | 1.685882 | 33.7176±3.14 |
| 6 | 2.1173 | 2.1358 | 2.1167 | 2.123267 | 2.678186 | 53.5637±3.98 |
| 24 | 2.6653 | 2.6527 | 2.6479 | 2.6553 | 3.450704 | 69.0141±4.17 |
| 48 | 3.1785 | 3.2072 | 3.192 | 3.192567 | 4.230821 | 84.6164±7.42 |
| 72 | 3.4669 | 3.4469 | 3.4215 | 3.4451 | 4.597503 | 91.950±6.18 |
