# Supplementary material for: Antibacterial effects of thyme oil loaded solid lipid and chitosan nano-carriers against Salmonella Typhimurium and Escherichia coli as food preservatives
Source: PLoS One. 2024 Dec 31;19(12):e0315543. doi: 10.1371/journal.pone.0315543 (PMC12140078; doi:10.1371/journal.pone.0315543)
Supplement: S3 Table — (DOCX) [file pone.0315543.s003.docx]

**Table S3.** Release kinetic parameters for TO-SLN and TO-CH based on mathematical models

| **Formulation** | **Zero order**  **R^2^** | **First order**  **R^2^** | **Higuchi**  **R^2^** | **Korsemeyer-peppas**  **R^2^** | **Hixson–Crowell**  **R^2^** |
| --- | --- | --- | --- | --- | --- |
| **TO-SLN** | 0.946 | 0.909 | 0.942 | 0.909 | 0.826 |
| **TO-CH** | 0.985 | 0.964 | 0.986 | 0.862 | 0.784 |
