# Supplementary material for: Antibacterial effects of thyme oil loaded solid lipid and chitosan nano-carriers against Salmonella Typhimurium and Escherichia coli as food preservatives
Source: PLoS One. 2024 Dec 31;19(12):e0315543. doi: 10.1371/journal.pone.0315543 (PMC12140078; doi:10.1371/journal.pone.0315543)
Supplement: S4 Table — (DOCX) [file pone.0315543.s004.docx]

**Table S4**. Log concentrations of inoculated *S. typhimurium* in fresh orange juice in presence of TO, TO-SLN and TO-CH in 6 days.

| **Log CFU/mL** | | | | | | | | | | | | | | | | |
| --- | --- | --- | --- | --- | --- | --- | --- | --- | --- | --- | --- | --- | --- | --- | --- | --- |
| **Concentrations** | **Oil** | | | | **SLN** | | | | **Chitosan** | | | | **control** | | | |
| **Days** | 0 | 2 | 4 | 6 | 0 | 2 | 4 | 6 | 0 | 2 | 4 | 6 | 0 | 2 | 4 | 6 |
| **MIC** | 3 | 0 | 1 | 4 | 3 | 0 | 0 | 1 | 3 | 0 | 0 | 0 | 3 | 5 | 6 | 7 |
| **1/2MIC** | 3 | 3 | 4 | 6 | 3 | 1 | 3 | 4 | 3 | 0 | 1 | 2 |  |  |  |  |
| **1/4MIC** | 3 | 4 | 6 | 7 | 3 | 2 | 4 | 5 | 3 | 1 | 1 | 2 |  |  |  |  |
