# Supplementary material for: Antibacterial effects of thyme oil loaded solid lipid and chitosan nano-carriers against Salmonella Typhimurium and Escherichia coli as food preservatives
Source: PLoS One. 2024 Dec 31;19(12):e0315543. doi: 10.1371/journal.pone.0315543 (PMC12140078; doi:10.1371/journal.pone.0315543)
Supplement: S1 Fig — a) Pure oil with MIC concentration. b) TO-SLN with MIC concentration. c) TO-CH with MIC concentration. d) control containing no antimicrobial agent. (DOCX) [file pone.0315543.s005.docx]

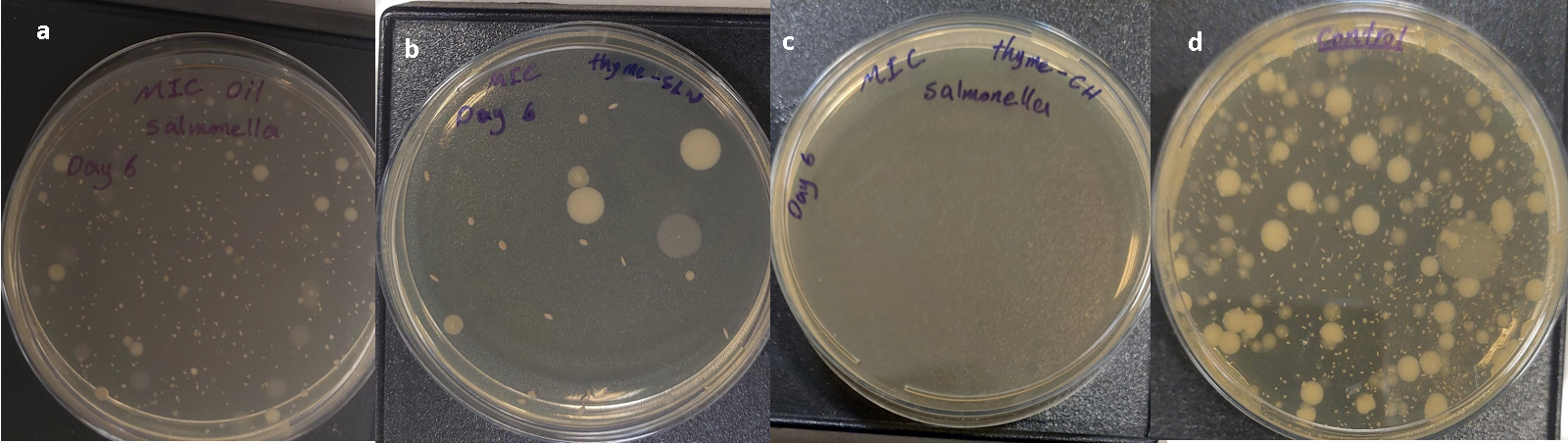


**Fig. S1**. Culture plates for counting the CFU existed in orange juice in day 6 of the *in vivo* study, a) Pure oil with MIC concentration. b) TO-SLN with MIC concentration. c) TO-CH with MIC concentration. d) control containing no antimicrobial agent.
