# Supplementary material for: Antibacterial effects of thyme oil loaded solid lipid and chitosan nano-carriers against Salmonella Typhimurium and Escherichia coli as food preservatives
Source: PLoS One. 2024 Dec 31;19(12):e0315543. doi: 10.1371/journal.pone.0315543 (PMC12140078; doi:10.1371/journal.pone.0315543)
Supplement: Attachment — Submitted filename: Response to referees.docx [file pone.0315543.s006.docx]

**Dear Editor in chief and Editorial Board of PLOS ONE journal**

Thank you for considering our manuscript “PONE-D-24-35651 Antibacterial effects of thyme oil loaded Solid Lipid and chitosan nano-carriers against *Salmonella Typhimurium* and *Escherichia coli* as food preservatives” for peer review and thanks to reviewers for their valuable time and comments. All comments are carefully addressed as follows. Due to the comments which needed some additional lab experiments, it took a relatively long time for us to do the revision, however, fortunately we didn’t pass the deadline.

Any further comments and questions are welcome by the research team.

Regards

Zahra Hesari

Abstract
Objective and conclusion must be clear

Authors: edited and highlighted.
Introduction
Introduction must be improving with focusing on the nanoparticles and thyme oils
Some previous studies finding should be highlights in the introduction section
Authors: some previous studies focusing of thyme oil nanoparticles (chitosan and lipid nanoparticles) has been added and highlighted in introduction.

Results and discussion
The results must be elobrate in more depth and discussion section must be improve

Authors: edited and highlighted. Results are discussed in a more depth in discussion section.
Conclusion
Conclusion section must be rewrite with highlights the findings of the study

Authors: conclusion has been completely revised.

Reviewer #1: The authors have investigated the antibacterial effects of thyme oil loaded solid lipid and chitosan nano-carriers against Salmonella Typhimurium and Escherichia coli as food preservatives. The study has highlighted important parameters which support the findings. The data is well presented by the authors. Some minor revisions are recommended for further improvements in the manuscript:
1. In section 2.1, mention the full form of abbreviation PVA.

Authors: Mentioned and highlighted.

2. In section 2.3.1, 2.3.2, 2.3.4 insert the references for the methodology followed.

Authors: References added and highlighted.

3. Mention the abbreviations of samples in the legend of table 1, table 2

Authors: Full description of sample names is added and highlighted to the abbreviations which were written before.

4. In the section 4, the year of publication should be mentioned with the references ‘Reis Mockdeci et al.’, Nemattalab et al., Al Hafi et al etc.

Authors: The year of publication is mentioned and highlighted.

5. The section 4 (discussion) could be improved by stating systematic explanation of the outcomes of each result along with the comparison with similar studies.

Authors: systematic explanation for results of physicochemical evaluations of nanoparticles (SLN and chitosan) have been added to discussion and highlighted.

6. A single format of writing (p < 0.05) should be used uniformly throughout the manuscript. In section 4, its mentioned as (Pvalue < 0.05) which differs from rest of the manuscript.

Authors: all *(p ˂0.05)* have been assimilated and highlighted.

7. The conclusion section could be written in a more scientific manner by explaining the pros of the outcomes and future scope of the study.

Authors: conclusion is completely revised based on practical application of TO-CH as food natural preservative and suggestions for future studies are mentioned.

8. Maintain same font style and font size as recommended in the author guidelines. The font style and font size are different for references than rest of the manuscript.

Authors: the font style and size of references has been coordinated with the rest of manuscript and highlighted.

Reviewer #2: The manuscript titled as Antibacterial effects of thyme oil loaded Solid Lipid and chitosan nano-carriers against Salmonella Typhimurium and Escherichia coli as food preservatives submitted to PLOS ONE with manuscript number PONE-D-24-35651 for possible publication is not suitable for the publication as such due to following points. It must be REJECTED.

• The antibacterial results are limited to in vitro experiments. The study could be strengthened by including in vivo evaluations to confirm its potential as a food preservative.

Authors: Based on referee’s comment The in vivo test was designed based on literature and performed and results are presented under the section of 3.5 and Fig.6.

• While the study compares TO-SLN and TO-CH, it doesn't explore why chitosan significantly enhances antibacterial effects compared to lipid-based systems. A deeper analysis of the underlying mechanisms would be valuable.

Authors: Added in discussion and highlighted.

• Long-term stability of the nanoparticle systems (especially for food applications) is not addressed, which is essential for practical implementation in the food industry.
Authors: As the long-term stability study needs at least 2 years (based on United State Pharmacopeia) of time, however we have started the stability studies for this and similar projects, the results will be used in scale up and production process.

• The diagrams need to be improved.

Authors: Diagrams in figures 1, 4 and 5 have been improved among visuality and resolution.

• Some sentences are lengthy and complex, which may obscure the key points so simplifying those sentences would improve clarity.

Authors: all the manuscript text was revised and edited sentences are highlighted.
• Discussion section is comprehensive and the comparison with existing literature is good but it can be improved by adding more information on how TO-CH nanoparticles achieve such high antibacterial activity and how and why is it more effective than others.

Authors: added and highlighted.

• Ensure the following: Avoid repetition of words, The terms in vitro, in vivo, names of the biological organisms should be italic.
Authors: all the manuscript text was revised based on this comment, all scientific names of the biological organisms (plants or bacteria) are provided in italic and highlighted. Also, the phrase “*in vitro*” which was mentioned once. Repetitions are deleted.

• Conclusion is very concise and summarizes only the findings of this study so emphasizing on the practical application of TO-CH as a natural food preservative and addressing the limitations of this study could strengthen the conclusion and make it more impactful.

Authors: conclusion is completely revised based on practical application of TO-CH as food natural preservative and limitations of current study is mentioned in final part of conclusion as suggestions.
