# Supplementary material for: Antibacterial effects of thyme oil loaded solid lipid and chitosan nano-carriers against Salmonella Typhimurium and Escherichia coli as food preservatives
Source: PLoS One. 2024 Dec 31;19(12):e0315543. doi: 10.1371/journal.pone.0315543 (PMC12140078; doi:10.1371/journal.pone.0315543)
Supplement: Attachment — Submitted filename: Response to reviewers3.docx [file pone.0315543.s007.docx]

**Dear Editor in chief and Editorial Board of PLOS ONE journal**

Thank you for considering our manuscript “PONE-D-24-35651 Antibacterial effects of thyme oil loaded Solid Lipid and chitosan nano-carriers against *Salmonella Typhimurium* and *Escherichia coli* as food preservatives” for third round of checking. All comments are carefully addressed as follows.

Any further comments and questions are welcome by the research team.

Regards

Zahra Hesari

1. Your ethics statement should only appear in the Methods section of your manuscript. If your ethics statement is written in any section besides the Methods, please delete it from any other section.

Authors: Ethic statement is now located in Methods section and is deleted from the final part of manuscript.

2. Before we proceed, we’ll require some additional clarification to ensure your submission adheres to the PLOS ONE Data Availability policy (<https://journals.plos.org/plosone/s/data-availability>).

A) Please clarify whether your manuscript and its Supporting Information files contain your minimal data set. The minimal data set is defined as the data set used to reach the conclusions drawn in the manuscript with related metadata and methods, and any additional data required to replicate the reported study findings in their entirety. This may include: a.) The values behind the means, standard deviations and other measures reported; b.) The values used to build graphs; c.) The points extracted from images for analysis (<https://journals.plos.org/plosone/s/data-availability#loc-minimal-data-set-definition>).

If your manuscript and its Supporting Information files currently contain the minimal data set for your study, we propose the following Data Availability statement to be published alongside your article:
"All relevant data are within the paper and its Supporting Information files."

B) If your manuscript and its Supporting Information files do not contain your minimal data set, please upload the minimal anonymized data set necessary to replicate your study findings as either Supporting Information files, or to a stable public repository. If you upload your data to a repository, please also provide the relevant URLs, DOIs, or accession numbers for other researchers to access your data directly. For a list of recommended public repositories, please see <https://journals.plos.org/plosone/s/recommended-repositories>.

Authors: our minimal data set including release values in different time points for both nanoparticles (Tables S1 and S2), release kinetic parameters (Table 3), Table and figures related to in vivo study of food preservation (table S4 and Fig S1) are provided in supporting information file.
